# Innovative and Healthier Dairy Products through the Addition of Microalgae: A Review

**DOI:** 10.3390/foods11050755

**Published:** 2022-03-05

**Authors:** Héctor Hernández, Maria Cristiana Nunes, Catarina Prista, Anabela Raymundo

**Affiliations:** LEAF (Linking Landscape Environment Agriculture and Food), Research Unit Instituto Superior de Agronomia, Universidade de Lisboa, Tapada da Ajuda, 1349-017 Lisboa, Portugal; hectorhh16@hotmail.com (H.H.); cprista@isa.ulisboa.pt (C.P.); anabraymundo@isa.ulisboa.pt (A.R.)

**Keywords:** microalgae, dairy products, bioactive compounds, legislation

## Abstract

In recent years, the development of healthier foods, richer in nutraceutical or functional compounds, has been in great demand. Microalgae are attracting increasing attention, as their incorporation in foods and beverages can be a promising strategy to develop sustainable foods with improved nutritional profiles and a strong positive impacts on health. Despite the increasing market demand in plant-based foods, the popularity of fermented dairy foods has increased in the recent years since they are a source of microorganisms with health-promoting effects. In this context, the incorporation of microalgae in cheeses, fermented milks and other dairy products represents an interesting approach towards the development of innovative and added-value hybrid products based on animal proteins and enriched with vegetable origin ingredients recognized as extremely valuable sources of bioactive compounds. The effect of the addition of microalgal biomass (*Chlorella vulgaris*, *Arthrospira platensis*, *Pavlova lutheri*, and *Diacronema vlkianum*, among others) and its derivates on the physicochemical composition, colorimetric and antioxidant properties, texture and rheology behavior, sensory profile, and viability of starter cultures and probiotics in yogurt, cheese and ice cream is discussed in the current work. This review of the literature on the incorporation of microalgae in dairy products aims to contribute to a better understanding of the potential use of these unique food ingredients in the development of new sustainable products and of their beneficial effects on health. Considering the importance of commercialization, regulatory issues about the use of microalgae in dairy products are also discussed.

## 1. Introduction

Microalgae are prokaryotic or eukaryotic photosynthetic microorganisms which have the ability to grow fast and to live under conditions not favorable to other species [1]. The biomass of these microorganisms is characterized for being a remarkable source of bioactive compounds and other products, which has led to a huge interest in their use in recent years [2,3]. Although approximately 10 million species of microalgae have been described in nature, so far only 50 of these species (mainly from *Arthrospira* (*Spirulina*), *Chlorella*, *Porphyry*, *Nannochloropsis*, *Haematococcaceae*, and *Dunaliella* genera) have been studied in detail in relation to their biotechnological use [4,5,6]. The nutritional composition of microalgae is very variable and depends enormously on the species and even within the species, depending on the growth conditions (composition of the medium, temperature and light regime) [7]. Table 1 shows the nutritional composition (protein, fat, carbohydrates, and minerals, among others) of the most studied microalgae species.

The use of microalgae covers different areas, involving many applications. In the food industry, microalgae are used in the development of vegetarian and vegan foods as a substitute for macronutrients of animal origin, namely proteins [8,9], essential fatty acids and vitamins [5]. Furthermore, these microorganisms are employed to enrich different products, such as biscuits, nutritional bars, juices, pasta, breads, and dairy products [10,11].

In addition, microalgal biomass has been used in animal feed due to its high content of protein and carbohydrates, beyond improving the immune response and fertility in animals [32]. Another promising application of microalgae is their use in the medical field, mainly as a source of health beneficial compounds with anti-cancer, anti-inflammatory or anti-hypertensive properties [8,33]. Moreover, the use of microalgae extends to areas such as biofuel or bioplastic production, due to their high lipid and protein content [34,35,36].

In recent years, the development of new products with improved nutritional, structural and sensory characteristics has been highly demanded by consumers. Food industries are continually exploring the potential of new ingredients, and some of these innovative ingredients are referred as functional or nutraceutical ingredients, since besides their nutritional value, they also have benefits on the human body, reducing the risk of disease or improving consumers’ health [4,11]. In the last decades, there has been a rising interest in finding natural innovative, nutritive and sustainable sources to produce nutraceutical ingredients. In this sense, microalgae are considered as one of the promising sources of functional food ingredients, resulting from their large amounts of bioactive compounds [37,38]. Various studies have shown the impact of fortification with microalgal biomass on several food products such as pasta, bread, and cookies, among others, evidencing the great potential of these microorganisms, even at low levels, in the production of healthy foods [12,16,18].

On the other hand, dairy products are considered an excellent nutritional source and are widely consumed by a large part of the world’s population [39,40,41]. In addition, these products are characterized by having great benefits on human health, for instance, positive effects on bones and teeth [42,43], hypertension [44], cardiovascular diseases [45,46], gastrointestinal health [47,48], muscle repair after exercise and the immune system [49]. Moreover, fermented dairy products have been attracting special attention, as beyond their nutritional and sensory profile they are a potential source of probiotics with a remarkable impact in the food–gut axis. However, in recent years the consumption of dairy products has been decreasing, since there is skepticism among the general consumers about the health effects of dairy foods, and also an increasing public concern about their sustainability since they are products of animal origin. For this reason, the search for new, healthier and more sustainable solutions is essential.

This review aims to show the latest research related to the use of microalgae and their derivatives in dairy products. In the literature, there are numerous review articles that show applications of microalgae in food in general, applications in the production of biofuels and applications in medicine. However, reviews related to the use of microalgae and their derivatives in dairy products are incipient. Therefore, this review article intends to highlight how the addition of microalgae affects the nutritional, rheological and sensory properties of this type of product.

## 2. Applications of Microalgal Biomass and Its Derivatives in Yogurt

Fermented milks as yogurt with probiotic microorganisms, presenting positive effects on health, are currently in high demand. Therefore, it is possible to find them in different types of markets everywhere worldwide. In order to increase their beneficial properties, there are some studies focused on the incorporation of different matrices rich in nutraceutical compounds in fermented milks, for instance, microalgal biomass or its derivatives (Table 2). In these studies, two ways to add microalgal biomass to yogurt were identified: the addition to the milk before the fermentation process (i), or the addition to the final product after the fermentation process (ii). The choice of each approach can affect the physicochemical, sensory or mechanical characteristics of the final product.

### 2.1. Changes in the Physicochemical Composition

The addition of *Arthrospira platensis* (also known as *Spirulina*, a cyanobacteria) biomass or its derivatives in yogurt formulations has been studied. Regarding the values of protein, fat and carbohydrates, the addition of microalgal biomass resulted in an increase in protein content. This behavior was expected, since *A. platensis* has a high protein content (around 70% in many cases [18,19]). Specifically, in the studies developed by Atallah et al. [50] it was observed that the protein values were 5.4% for the control sample and 6.2% for the yogurt fortified with 1% *A. platensis* added before the fermentation process. Similar results were reported by Silva et al. [51] in yogurts with *A. platensis* added after the fermentation process. No significant differences were found in fat and carbohydrate content between the controls and the fortified yogurt samples, due to the lowest concentration of fat and carbohydrates in *A. platensis*.

Phycocyanin, a pigment extracted from *A. platensis,* has also been intensively studied. This pigment can be used as a functional ingredient, and also for its therapeutic properties: anti-cancer, anti-inflammatory, antioxidant, and nephroprotective [58]. In the food industry, this phycophiliprotein has been used as a natural dye due to its bluish color, being a good alternative to toxic and artificial coloring agents [55].

In the study developed by Mohammadi et al. [55], pH values in phycocyanin-fortified yogurts were reported. Specifically, it was observed that as the concentration of phycocyanin increased (2, 4, and 8%) the pH values of the fortified yogurt samples increased (4.74, 4.80 and 4.92), showing values higher than the control (4.4). This increase in pH should have resulted from the phycocyanin pH (6.55). In addition, the effect of storage on the pH of yogurts was also studied, indicating a slight increase in pH in all control and fortified yogurts during storage [55], and likely the production of some metabolites, such as amino acids and bacteriocins at the end of the storage time, may have impacted the increase in pH.

### 2.2. Changes in Color Parameters

Color is an important characteristic in yogurt, being very associated with the acceptability of this product. Different studies have shown the effect of the addition of microalgae on the color of the final products by using the CIELab scale (L*, degree of lightness; a*, degree of redness and greenness; b*, degree of yellowness and blueness). Barkallah et al. [56] evaluated the color parameters of *A. platensis*-fortified yogurts and reported that samples enriched with 0.25% *A. platensis* powder were characterized by having the lowest values of a* and b*, which indicates that the color samples changed from yellow to greenish. This is due to the high concentration of chlorophyll in *A. platensis*. Robertson et al. [54] also observed a similar trend in the color of samples of yogurt fortified with 0.25% and 0.5% *Pavlova lutheri* at 28 days of storage.

On the other hand, factors such as acidity and pH can also affect the color of yogurt in storage. The phycocyanin present in *A. platensis* can be used as a pigment in foods with a pH above 4.0 if it is not exposed to heat or light [59,60]. The results found by Mohammadi et al. [55] are in agreement with this information, since the values of L*, a*, and b* in yogurts enriched with 2%, 4% and 8% of phycocyanin with pH values above 4.0 showed no significant differences during 28 days of storage. Due to the instability of the pigments present in *A. platensis*, Alizadeh et al. [57] studied the color changes in yogurts with 0.25% and 0.5% *A. platensis* with the addition of Zedo gum (an exudate from *Amygdalus scoparia*), demonstrating that there was a stabilization of microalgal biomass because of the interaction between milk proteins and the added anionic polysaccharide. Due to acidification caused by *Lactobacillus*, the pH of the medium took values lower than the PI of the proteins, which favored the interaction with the gum [61]. 

All of the aforementioned studies have the common conclusion that *A. platensis* pigments can be used as natural colorants in fermented milks, resulting in products with innovative colors that can contribute to broadening the market’s offerings.

### 2.3. Effect on Antioxidant Properties

The addition of microalgae to yogurt has the benefit of incorporating different bioactive compounds with antioxidant properties, such as beta-carotene, astaxanthin and lutein [62]. Inherently, yogurt has an interesting antioxidant capacity, due to the presence of sulfur-containing amino acids, carotenoids, zinc, selenium and some enzymes produced in the fermentation process [63]. Nevertheless, some studies have reported an increased antioxidant capacity of yogurts when fortified with microalgal biomass. Silva et al. [51] observed that the antioxidant capacity increased by 150% in yogurts fortified with 1% *A. platensis*, when compared to the control (yogurt without microalgae addition). Likewise, Atallah et al. [50] and Alizadeh et al. [57] also studied the antioxidant activity of yogurt enriched with 1.0 and 0.5% *A. platensis* powder and observed a sharp rise in the antioxidant activity corresponding to 200.02% and 110.88% for each treatment. This results from the presence of chlorophyll, carotenoids, and phycocyanin in *A. platensis* biomass, the release of which can be increased by the interaction with the lactic acid bacteria present in the fermented dairy. Therefore, it is possible to state that the addition of microalgae to yogurt can provide health benefits to consumers.

### 2.4. Changes in Growth of Starter and Probiotic Cultures

The incorporation of microalgae into yogurt provides a favorable growth environment for microorganisms due to their ability to supply many nutrients for the growth and viability of lactic acid and probiotic bacteria. Substances such as exopolysaccharides, adenine, hypoxanthine, free amino acids, minerals and vitamins are provided by different species of microalgae [52,64,65]. The addition of microalgal biomass also influences the buffer capacity of the medium, which can affect the viability of probiotic microorganisms in the fermentation process, although, depending on the type of microalgae used, this parameter may vary. Treatments with lower buffer capacity usually cause a stronger pH decrease during the fermentation process and storage, resulting in a pH drop shock to probiotic bacteria which leads to a reduction of their viability and more reduced growth times until the end of fermentation [66,67]. In the study developed by Beheshtipour et al. [52], the effect of incorporating *Chlorella vulgaris* and *Arthrospira platensis* in the fermentation process of yogurts was studied and the results indicated that the fortification with *A. platensis* at the concentration of 0.25, 0.50 and 1.0% showed a greater buffer capacity compared to the treatments enriched with *C. vulgaris* at the same concentration values; however, the large amount of nutrients supplied by *C. vulgaris* compensated its lower buffer capacity, resulting in non-significant differences in the viability of probiotics in samples with both microalgae. Alizadeh et al. [57] also studied the incorporation of *A. platensis* in yogurts and observed that in microalgae-enriched samples ranging from 0.13 to 0.5% there was a higher growth rate of starter microorganisms with values above 10^8^ CFU/g, compared to control (yogurt without microalgae added) with values above 10^6^ CFU/g, resulting in increased lactic acid production during the fermentation process.

### 2.5. Effects on Syneresis, Texture and Viscosity

Dairy products such as yogurt are studied based on three properties that affect their quality: syneresis, texture and viscosity [68]. Syneresis, also known as serum separation, is considered an undesirable property in fermented products such as yogurt and it is generally strongly related to changes in the casein-gel network [69]. Some studies report that a higher percentage of protein and fat globules in milk results in lower levels of syneresis [56,70]. The addition of microalgal biomass can affect syneresis on the final product. The increase in the amount of *A. platensis* can reduce the yogurt syneresis rate due to the high concentration of carbohydrates and proteins of the microalgae, and thus high water holding capacity. In addition, Robertson et al. [54] reported syneresis values of 66.1 and 68.2% for yogurt fortified with 0.25% *Pavlova lutheri* and the control sample, respectively, which indicates a reduction in this property. Therefore, it is suggested that microalgal biomass works as a stabilizer in yogurts to decrease syneresis, which has a positive impact on the characteristics of the final product.

Supplementing yogurt with microalgae and their derivatives can also affect the texture properties of yogurt. Bchir et al. [58] reported that in yogurts with concentration of *A. platensis* above 0.3%, there was an increase in the gel hardness with values greater than 0.67 N when compared to the control sample (0.60 N), which indicated the reinforcement of the three-dimensional network of the gel. The increase in hardness could result from the acidification of the lactic bacteria, which strengthened the gel network through the absorption of water and also from the high protein content of the algae. An increase in protein concentration can improve the interaction of these biomolecules, resulting in a firmer gel [71]. However, other studies such as those developed by Barkallah et al. [56] indicated that increases in the percentage of *A. platensis* addition can lead to lower firmness values due to the breaking up of the three-dimensional network.

The yogurt rheology behavior plays a fundamental role in obtaining new formulations, as it is the result of the formation of a three-dimensional network composed of casein, denatured whey proteins and fat globules [50,72]. Yogurt viscosity depends on several factors, such as the species of starter cultures, concentration of starter microorganisms, handling, addition of stabilizers and storage [54]. In addition, one of the most relevant properties is the apparent viscosity, which is dependent on the level and the type of the microalgae incorporated. Higher amounts of added microalgae can result in an increase in apparent viscosity. In a study developed by Bchir et al. [58] it was observed that in samples fortified with 0.5% of *A. platensis* the apparent viscosity value at a shear rate of 200 rpm was 903 mPa.s, while for unfortified samples the value obtained was 700 mPa.s (*p* < 0.05). These differences in viscosity can be attributed to the enhanced protein concentration in fortified yogurts, which may be responsible for the increase in their viscosity, contributing to an increase in the degree of structuring of the system [73]. However, authors such as Mohammadi et al. [55] reported that the incorporation of phycocyanin resulted in a decrease in apparent viscosity. According to this study, this could have occurred for two reasons: (i) the phycocyanin may have caused the breaking of the three-dimensional network, reducing the viscosity and therefore the surface tension, and (ii) in the phycocyanin incorporation process, a high-speed stirring step was carried out, resulting in lower viscosity values than the control. 

### 2.6. Effect on Sensory Properties

Due to the highlighted sensory attributes such as fishy taste in many aromatic compounds and pigments, it is expected that the incorporation of microalgae and their derivates in yogurt have a significant effect on the sensory characteristics of this type of product. The addition of 0.25 and 0.5% (*w*/*v*) of *P. lutheri* significantly affected the colorimetric properties of yogurt due to the presence of carotenoids, chlorophyll, beta-carotenes, and fucoxanthin, among others. This result was corroborated in sensory analysis by 40 untrained panelists, who did not receive the fishy taste and yogurt flavor well, and scored poorly color the characteristics and the overall acceptability of the fortified yogurt when compared to the control (yogurts without microalgae) [54]. Similar results were obtained by Bchir et al. [58] who reported that the highest percentage of *A. platensis* addition (0.5% *w*/*w*) resulted in a very greenish color and microalgal sedimentation in yogurt; these factors certainly induced the low score obtained, in terms of appearance and overall acceptability (4.7 and 4.87, respectively, on a scale from 1 to 7). Indeed, yogurts fortified with 0.5% of *A. platensis* had all sensory attributes with low scores. Atallah et al. [50] also reported that yogurt supplemented with 1% *A. platensis* showed the lowest scores of all sensory attributes (40.33, 38.33, 6.83 and 85.50% for flavor, body and texture, appearance and total acceptability), when compared to yogurts fortified with whey protein and sodium caseinate. In fact, some authors suggest the use of microencapsulation techniques in order to improve the sensory characteristics of products supplemented with substances of marine origin [51,69,70,74,75]. 

On the other hand, concerning phycocyanin, one of the important health beneficial compounds from microalgae, the sensory analysis of phycocyanin-enriched yogurts performed by 15 analysts showed a higher consistency score compared to the control, but these differences were not statistically significant. In addition, yogurts fortified with 4% of phycocyanin were chosen by the panelists as having the highest overall acceptability [55].

In general, it can be concluded that the incorporation of microalgae in yogurt is a complex process, which must take into account many properties that can affect the sensory quality of the final product. Therefore, in order to circumvent these issues and increase the overall acceptability of fermented dairy products enriched with microalgae, it is necessary to carry out further studies and find strategies to minimize the algae flavor and aroma, which is not expected in this type of product.

## 3. Applications of Microalgal Biomass and Its Derivatives in Ice Cream

Ice cream is a dairy product that has a great consumption worldwide because of its nutritional properties and refreshing effect, especially in warmer weather days. This product is made from milk, sweeteners, stabilizers, emulsifiers, flavoring and coloring agents [76]. Some studies have shown the use of microalgal biomass in ice cream due to the high presence of pigments and compounds with stabilizing roles. Since food dyes have become common in the food production industry, there has been a debate about the harmful effects of artificial food colors. Therefore, the use of natural and functional pigments has increased in the recent years [76]. Table 3 presents some research on the incorporation of microalgae and their derivatives in ice cream.

### 3.1. Changes in the Physicochemical Composition

Some studies report that the addition of microalgae to ice cream affects its physicochemical characteristics. Properties such as pH and acidity of the ice cream mix can vary due to the increase in solids-non-fat caused by the incorporation of microalgal biomass. Malik et al. [71] reported an acidity increase from 0.1% to 0.25% lactic acid and a pH reduction from 6.20 to 6.11 in the ice cream mix when the *A. platensis* concentration ranged from 0.075 to 0.3% *w*/*w*. This can occur due to the buffer capacity of *A. platensis* and its physicochemical composition. Furthermore, in the research developed by Balensiefer et al. [77], it was observed that the addition of 1% pure or microencapsulated *A. platensis* to ice cream resulted in an increase in protein and fat content, when compared to non-enriched samples. This was expected since microalgal biomass from *A. platensis* has a high protein and fat content [77]. Agustini et al. [78] also reported a similar behavior in samples enriched with this strain. 

Another very important characteristic in the quality and freezing properties of ice cream is the melting time. This parameter depends on different factors, such as total solids, ice crystal sizes, quantity and size of fat globules, presence of stabilizers, emulsifiers, and storage [81,82]. Agustini et al. [78] reported melting times of 24.26 and 28.08 min/10 g for 0.6 and 1.2% *A. platensis*-fortified ice cream, respectively, and 21.38 min/10 g for the control. This reduction in the melting time could be a consequence of the high fat content of the enriched samples that decreased the heat transfer rate, resulting in higher melting times [77]. On the other hand, when microalgae derivatives such as pigments are incorporated, a different behavior can be observed in the melting time of ice cream. Rodrigues et al. [80] reported that the use of 0.5% *w*/*v* phycocyanin as a substitute for stabilizers in ice cream resulted in shorter relative first drop times (below 10.0 s.g^−1^) when compared to the control (13.0 s.g^−1^), which indicates that this pigment does not improve the stabilizing capacity of the ice cream.

Furthermore, the overrun, defined as the increase in ice cream volume due to the incorporation of air, also plays an important role in the ice cream quality [82]. The addition of microalgal biomass may influence this parameter. Agustini et al. [78] observed that overrun values in 0.6 and 1.2% *w*/*w A. platensis*-enriched samples were higher (35.25 and 37.62%, respectively) than the value found in the unfortified product (33.99%). One reason for this trend might be the techno-functional properties of the microalgae proteins. *A. platensis* has an emulsifying capacity of 1.13 mL of fat/g of protein, a foaming capacity of 207% and a foam stability of 27% [83], and it is possibly responsible for a greater amount of air incorporated into the mixture and consequently for an increase in its volume. Malik et al. [71] also found a similar behavior in ice cream supplemented with this microalgal biomass.

### 3.2. Changes in Color Parameters

The usage of microalgae as coloring agents extends to products such as mayonnaise, yogurt, pasta and chewing gum [56,76,84,85]. In dairy products such as ice cream, some studies have been carried out. The presence of natural dyes such as phycocyanin, chlorophyll, fucocyanin, fucoxanthin and anthocyanins in microalgae can affect the color of the final product [86,87]. Durmaz et al. [76] observed color changes in ice cream enriched with 0.1, 0.2 and 0.3% *P. cruentum* biomass resulting in samples with a pinkish hue. The cause of this color change was associated with the presence of phycophiliproteins, namely fucocyanin, a water-soluble, red pigment. In the same study, it was observed that the color parameters L*, a* and b* were differently affected by the concentration of microalgae. While L* values were not significantly affected by the concentration of *P. cruentum*, the b* and a* values increased from to 8.40 to 8.73 and 6.20 to 11.41, respectively, with the increment of the microalgal biomass content, resulting in greater intensity of red color. Storage color stability is also an important parameter in ice cream development. In a study developed by Campos et al. [79], the use of phycocyanin as a natural coloring agent in ice cream was studied. The results revealed that the value of the total color difference parameter (ΔE*) in samples with 0.025% phycocyanin was 4.50 and 9.46 after 14 and 182 days, respectively, whereas the control samples showed values of 2.28 and 15.20, respectively, indicating that the difference in ΔE* values in samples with phycocyanin were lower, which demonstrates the good stability of this natural pigment during storage. Moreover, factors such as pH also affect color stability. Phycocyanin is stable over a pH range of 5, and values above or below this range can cause color loss [87]. Bearing in mind that ice cream is considered a neutral product (pH 6–7) [71,88], phycocyanin is expected to have a promising use as a natural coloring agent in this product.

### 3.3. Effect on Rheology Parameters

The rheology parameters of the ice cream mix play a fundamental role when keeping all components in a homogeneous state. The addition of microalgal biomass could affect the rheology behavior of ice cream. Malik et al. [71] observed that an increase in the concentration of *A. platensis* as a substitute for stabilizers in ice cream resulted in a reduction in viscosity. The authors explain that *A. platensis* has a low water absorption capacity (1.45 g of water/g of protein) when compared to traditional stabilizers, for instance casein (2.5 g of water/g of protein). For this reason, the ability to form a gel structure with water was reduced when microalgae were added, and consequently, the sample viscosity decreased [83].

The flow index (*n*) and consistency index (*K*) parameters can be affected by the incorporation of microalgal biomass. Lower *n* values indicate a high shear thinning behavior (i.e., viscosity decreases with increasing shear rate). The shear thinning behavior is important in the processing and the sensory characteristics of ice cream, since the higher values of this property allow an easier pumping and mixing of the mix and consequently a reduction in the energy consumption of the process [89]. Concerning this parameter, it was reported that ice cream mix fortified with increasing *P. cruentum* concentration (0.1, 0.2 and 0.3% *w*/*w*) presented lower flow index (below 0.324) when compared to the control sample (0.361) [76]. However, it is important to take into account the type of microalgae used, since the presence of macronutrients in addition to pigments and other bioactive compounds can result in an increase in the flow index. Likewise, *K* values could be affected by the composition and amount of microalgae used. For instance, the biomass of *P. cruentum* contains stabilizing agents such as membrane polymers, extracellular polysaccharides and sulfated polysaccharides that can increase the consistency index in ice cream enriched with this species. 

### 3.4. Effect on Sensory Properties

Sensory characteristics such as color, aroma, flavor, taste and texture may be affected by the incorporation of microalgal biomass in ice cream. Durmaz et al. [76] reported that the addition of *P. cruentum* resulted in a positive effect on ice cream color due to the pink color coming from the microalgae natural pigments. However, according to the perception of panelists, excessive amounts of microalgae can negatively affect sensory attributes such as flavor, aroma, and overall acceptability. The flavor of ice cream depends mainly on the fat in the milk. The addition of 0.3% of *P. cruentum* originates a fishy aroma undesirable in ice cream. In the same study, *N. oculata* and *D. vlkianum* were also analyzed, and the results showed that the increase in the amount of added microalgae negatively affected the ice cream flavor because according to the panelists, the bitter taste was stronger. These results are in agreement with those obtained by Fradique et al. [84] in pasta and Barkallah et al. [56] in yogurt, who observed low scores for the flavor, aroma and general acceptability of the fortified samples.

Some approaches propose the usage of a low-temperature and vacuum deodorization processes in order to remove compounds from undesirable aroma generators in microalgal biomass, and likewise the addition of masking or encapsulating compounds that reduce the perception of these aromas. However, in the study carried out by Balensiefer et al. [77], it was observed that the addition of microencapsulated *A. platensis* within arabic gum or maltodextrin had no significant effects on aroma scores, when compared to samples with un-encapsulated microalgae. Indeed, additional studies are needed to determine the most appropriate percentage of microalgae addition without drastically affecting the sensory properties of the final product.

## 4. Applications of Microalgal Biomass in Cheeses

Cheese is a highly consumed dairy product around the world with a remarkable variety of aromas and shapes associated with the interaction of milk proteins, carbohydrates and fat, and the effect of bacteria in raw milk initiates microorganisms and probiotics [90]. In order to improve the sensory, nutritional and functional characteristics of cheeses, in recent years, studies have been carried out on the incorporation of microalgal biomass. Table 4 presents some of more recent research. 

### 4.1. Changes in the Physicochemical Composition

The incorporation of microalgal biomass can affect the physicochemical properties of cheeses. Mohamed et al. [91] observed an increase in protein values ranging from 14.38 to 15.70% and a reduction in pH values from 5.42 to 5.39 in cheese analogue fortified with *C. vulgaris* at 1, 2 and 3% *w*/*w*, when compared to the control (13.56% protein, pH 5.80). This is explained as follows: cheese analogue samples were manufactured using as the base cheese an acid and unsalted curd (43.01% protein and pH 4.7), whereas the control was manufactured with a mixture of Ras cheese (22.6% protein) and ripened Cheddar cheese (25.47% protein). Therefore, the acid curd and its higher protein content had an effect on decreasing pH values and also had an effect on increasing protein levels in the supplemented samples. In addition, protein content in *C. vulgaris* biomass also contributed to increased protein values. A similar behavior was also observed by Muir et al. [97] and Cunha et al. [98] in other cheese analogues. Likewise, Agustini et al. [78] indicated an increase in the protein content (above 22%) in soft cheese enriched with 1.5% *A. platensis* when compared to the control sample (3.8%).

The effect of storage on the pH of cheese has also been studied. Golmakani et al. [95] observed that after 15 days of storage, the pH values of feta-type cheeses fortified with 0.5, 1, and 1.5% *A. platensis* were 4.81, 4.82 and 4.86, respectively, while the control sample was 4.6. This behavior may be related to the buffer capacity of *A. platensis* caused by its physicochemical components such as proteins, peptides and amino acids. Furthermore, the starter bacteria could have been in a late log phase or an early lag growth phase in the acidification process, and consequently, the maturation process may have caused the formation of peptides, resulting in pH increases [52]. On the other hand, melting is a very studied property in cheese analogues. Mohamed et al. [94] reported a decrease in melting index values (91, 88 and 86 mm) of cheese analogues enriched with *A. platensis* with the increase of algae concentration (1, 2 and 3% *w*/*w*), when compared to the control (111.0 mm). This could have occurred due to that the network in the control sample was weaker for being obtained by enzymatic coagulation, whereas the treatments were made with acid casein, which is characterized by having a lower melting rate [99].

Finally, the oil separation index in processed cheeses can also be affected by the incorporation of microalgae. This physicochemical characteristic largely depends on the state of the fat and protein in the emulsion, the type and quantity of materials used in the formulation, the cooking time and temperature, type of emulsifying salt, and the final pH of the product, and it is used to measure the emulsion stability in cheese [100]. The addition of 3% *C. vulgaris* to processed cheese analogues resulted in a decrease in oil separation rates (17%) when compared to the control sample (25%). One of the factors that may have influenced this behavior is the nutritional composition of *C. vulgaris*, since its biomass is rich in proteins and carbohydrates, which resulted in more stable emulsions in fortified samples. Mohamed et al. [94] also observed a similar trend in processed cheese enriched with *A. platensis*.

### 4.2. Changes in Color Parameters

In dairy products such as cheese, there is a direct relationship between color and consumer acceptance, therefore changes in the color of this traditional product can affect their sensory characteristics and consequently the buying intention [101]. The incorporation of microalgae in cheese results in the coloration of this product due to the presence of a varied number of pigments in the microalgal biomass. Mohamed [96] developed a study on the incorporation of small *A. platensis* grains in Kareish cheese, a very popular product in Egyptian cities, and the results showed that there were changes in the color parameters L*, a* and b* when the algae concentration was increased. Samples containing 1.5% *A. platensis* showed a stronger greenish color, indicating a reduction in a* values ranging from 1.02 for control sample to -10.69 for supplemented sample. These color changes are caused by pigments such as chlorophyll and phycocyanin present in the microalgal biomass. A similar trend was indicated by Golmakani et al. [95] in feta-type cheese fortified with this microalgae species. In addition, the authors reported that after 60 days of storage, color changes were negligible for each treatment.

### 4.3. Effect on Textural Properties

Food texture is an essential property of the cheese, and is well appreciated by consumers. The addition of microalgae in cheeses can affect the texture parameters of this dairy product [102,103]. The incorporation of *C. vulgaris* in processed cheese analogues resulted in higher firmness values [91]. This species has a high level of protein and therefore plays an important role in the water absorption capacity, which promotes increased firmness in cheese [104]. This trend was also found in the studies developed by Mohamed [98], who showed that the incorporation of *A. platensis* in Kareish cheese resulted in increased firmness values (4.4 N for the control sample and 4.9 N for the sample enriched with 1.5% *A. platensis*). Fradique et al. [84] and Khemiri et al. [37] also indicated similar behavior in pasta and bread, respectively. Therefore, the use of microalgae to improve the textural properties of cheese is a promising field for the development of new products.

### 4.4. Effect on the Antioxidant Activity

Antioxidants are compounds that help combat cell and DNA damage that develops into cancer, coronary heart disease or other chronic diseases. Dairy products such as cheese are rich in antioxidant compounds and fortifying them with the incorporation of microalgae can further improve their antioxidant activity. According to Tohamy et al. [92], the addition of *C. vulgaris* (4% *w*/*w*) in processed cheese resulted in an increase in antioxidant activity going from 54.85% in control samples to 68.3% of Radical Scavenging Capacity in samples enriched with microalgae. This difference can be attributed to the composition of *C. vulgaris*, which is rich in several antioxidants such as lutein, chlorophyll and selenium [105]. In the same study, the authors indicate that the cooking temperature of processed cheese may also have contributed to the difference in antioxidant capacity, since high temperatures (90–150 °C) affect the antioxidant activity. Mohamed et al. [94] reported an antioxidant capacity above 57% in processed cheese analogues enriched with 3% *A. platensis* and 5.52% for the control sample, indicating that the antioxidant activity was improved. *A. platensis* has different antioxidant compounds such as phycocyanin, tocopherols, and phenolic compounds, which caused this increase.

### 4.5. Changes in the Sensory Properties

The study of sensory parameters greatly contributes to the possible marketing of cheese since it shows a perspective of potential consumer acceptability. The incorporation of microalgal biomass in cheeses results in changes in organoleptic parameters, for instance, high concentrations of microalgae *C. vulgaris* (≥4%) can result in cheeses with a granular texture that is not well received by consumers [92]. Properties such as flavor can also be affected by the incorporation of microalgae in cheese. Bosnea et al. [93] reported that addition of *A. platensis* to Greek soft cheese at 1% level resulted in having a bitter aroma and taste due to the characteristic of microalgae. In addition, the storage effect must also be considered when studying the sensory properties of microalgae-enriched cheeses. In a study developed by Golmakani et al. [95], feta-type cheese fortified with 0.5, 1.0 and 1.5% *A. platensis* was produced, and the results of the sensory analysis indicated that the bitterness of the samples was more accentuated with an increasing amount of microalgae and an increase in storage time. This can be attributed to the physical characteristics of the microalgae, as well to the effect of proteolysis that occurred during storage, which may have caused the accumulation of peptides that gives the cheese a bitter taste [106].

In order to reduce the algae-like aroma, some researchers have proposed different methods such as heating, enzymatic hydrolysis, inclusion of beta-cyclodextrins, fermentation and solvent extraction [107]. However, more studies on the sensory properties of microalgae-enriched cheeses should be developed, and consequently the best method should be found for removing undesirable attributes in the final product.

## 5. Other Dairy Products

The effect of the incorporation of microalgae has also been studied in some dairy products not included in the aforementioned categories, for instance: fermented milk powder, kefir, buttermilk beverage and Labenah (a product originated in the Middle East considered as a hybrid mixture between cheese and yogurt). 

Vlasenko et al. [108] developed a fermented beverage based on buttermilk enriched with *A. platensis* at 1.0, 1.5, 2.0 and 2.5% (*w*/*w*) and the results indicated that treatments with percentages of microalgal biomass lower than 2% showed acceptable acidity values (75–80 °T); however, treatments with algae concentrations greater than 2% resulted in very high acidity values (87–90 °T). Due to a higher substrate concentration, lactic acid bacteria were able to produce a higher amount of lactic acid during fermentation [109]. The fat content value for all treatments and the control was 0.4% (*p* > 0.05).

Martelli et al. [110] reported the effect of adding *A. platensis* (0.25 and 0.5% *w*/*v*) and *Lactobacillus bulgaricus* and *Streptococcus thermophilus* in reconstituted fermented milk powder (10% *w*/*v*), and it was observed that there was a significant decrease in the pH values (4.3 and 4.1) when the microalgae concentration was increased. This behavior is similar to that reported by Varga et al. [111], who observed a decrease in pH values in milk containing *A. platensis* and inoculated with *S. thermophilus* and *L. bulgaricus* when the algae concentration was increased. Likewise, the authors studied the effect of microalgal biomass addition on rheological behavior in terms of flow index (n) and consistency index (K). The results showed that there was a decrease in K values (2.56 and 1.77 Pa.s^n^) with the addition of 0.25 and 0.5% *A. platensis*, related to a reduction in the consistency when compared to the control sample (product without microalgae). The values of flow index (n) were increased with microalgae addition, ranging from 0.366 to 0.439, revealing a reduction in the shear thinning behavior.

Mohamed et al. [112] developed a high-quality protein Labenah enriched with *A. platensis* (0.5% *w*/*w*), and the results indicated that there was a significant increase in protein content (13.08% *w*/*w*) when compared to a control sample of 10.60% *w*/*w*. Microalgal biomass of *A. platensis* is known to have high levels of protein [18,19], and therefore its incorporation in Labenah resulted in an increase in the content value of this macronutrient. A similar trend was observed by Laela et al. [113], who reported a protein content of 5.53% in kefir fortified with 2% *A. platensis* compared to 4.02% in the control sample. In addition, the effect of storage on the acidity level of Labenah was also evaluated. Mohamed et al. [112] observed that there was an increase in the acidity values of this product, going from 2.2% (day 0) to 2.8% lactic acid (day 27) in samples enriched with *A. platensis* (0.5% *w*/*w*). This increase was more significant than in the control sample (from 1.7 to 1.8% lactic acid on day 0 and 27, respectively), which indicates that lactic acid production was beneficially influenced by the addition of microalgal biomass.

## 6. Regulation Issues of Microalgal Biomass or Derivates in Dairy Products

Microalgal biomass and its derivatives are added to dairy products due to their functional and nutraceutical properties, as discussed in the present review. However, fortification of foods with novel ingredients is regulated by different authorities around the world according to Table 5, namely the European Food Safety Authority (EFSA), the Food and Drug Administration (FDA), the Food Safety and Standards Authority of India (FSSAI) and the Australia New Zealand Food Authority (ANZFA), in order to ensure that these added ingredients are safe. The safety of products must be evaluated before their launch to the market, but restrictive regulatory requirements can delay their commercialization.

Regarding the incorporation of microalgal biomass and its derivatives in dairy products, the European Union (EU), through EFSA regulation 258/97 [114], established that microalgae used as food ingredients before May 15, 1997 are not considered as “novel food” or “novel ingredient” and can be added in unlimited concentrations in all dairy products. For this reason, the incorporation of microalgal biomass or derivatives of *Arthrospira platensis*, *Chlorella vulgaris*, *Chlorella luteoviridis* and *Chlorella pyrenoidosa* has been studied in greater proportion [77,78,91,92,120]. In addition, foods fortified with microalgae may have nutritional claims such as “high protein” or “source of protein” as defined in regulation (EC) 1924/2006 [121], since these ingredients have been proven to have a high protein content [18,19]. Moreover, the incorporation of *E. glacilis* and *Ulkenia sp.* Derivatives in dairy products has been approved by EFSA according to the regulation 2017/2470 [115] with maximum levels shown in Table 5.

On the other hand, in the United States of America (USA) it is very common for substances that are recognized as safe (GRAS) to be added to foods. GRAS status is granted by the FDA and depends on a series of rigorous scientific procedures that demonstrate the ingredient’s safety in foods. The FDA has received several GRAS notices related to the use of microalgal biomass in a wide variety of products including dairy products. For instance, the biomass of *E. gracilis* (GNR 697), *A. platensis* (GNR 417) and *C. protothecoides* (GNR 519) can be incorporated into milk and dairy products with different concentrations, according to Table 5 [116].

A very common feature among all current regulations is the low value of the maximum levels of incorporation (no more than 2 g/100 g in most cases) of microalgal biomass in all dairy products, which has not allowed a further development of the food industry dedicated to the production of milk products fortified with these ingredients.

## 7. Final Remarks

The addition of microalgae to dairy products results in changes in the physicochemical properties of these foods, namely the protein content and pH. According to the literature review, increases in the percentage of added algae reflect a decrease in yogurt syneresis, which is considered a favorable effect in this type of product. In ice cream, fortification with microalgal biomass allows a reduction in melting time as well as the replacement of artificial dyes by natural dyes present in microalgae, and according to several studies, the stability of these pigments during storage is very high. Cheese enriched with microalgae has a greater antioxidant capacity, due to the high phenolic compounds and carotenoid contents in the microalgal biomass. Despite these benefits, the sensory attributes of dairy products are negatively affected by the incorporation of microalgae, which is a major constraint, making it necessary to carry out further studies in this area in order to create products that are well received by the consumer. Despite this, based on the information reported in the literature, it is likely that the fortification of dairy products with microalgae or its derivatives is an approach that has great potential to obtain more sustainable and healthier foods. The clarification of the influence of microalgae in the growth, the viability and the secondary metabolites of lactic acid bacteria and other probiotic microorganisms, and also their impact in flavor and spoilage during maturation and storage, could be of great value for the microalgae and dairy industries.

## Figures and Tables

**Table 1:** Physicochemical composition of the most studied species of microalgae.

Physicochemical Composition	Species						
	*Chlorella vulgaris* ^1^	*Nannochloropsis gaditana* ^2^	*Arthrospira platensis* ^3^	*Auxenochlorella protothecoides* ^4^	*Euglena gracilis* ^5^	*Dunaliella bardawil* ^6^	*Tetraselmis chuii* ^7^
Protein (% dry matter)	12–44	18–50	50–70	6–43	41–47	29–31	11–46
Lipid (% dry matter)	22–46	10–17	8–9.3	7–59	13–23	10–19	0.3–23
Carbohydrate (% dry matter)	24–39	15–31	13–48	15–35	34–43	11–12	30–54
Pigments							
Lutein (mg/kg)	0.2–5	n.r	n.r	n.r	n.r	4.2–6.7	624
Chlorophyll (mg/L)	6–18	0.3–2.3	5–14	0.1–4	1–5.3	7.9–9.1	353–400
Phycocyanin (mg/mL)	n.r	n.r	0.5–2.3	n.r	n.r	n.r	n.r
Beta-carotene (mg/g)	n.r	0.1–2.9	n.r	0.1–1.1	0.1–52	0.8–1.5	0.1–1
Vitamins (mg/kg)							
B2	20–34	25–62	34–81	n.r	n.r	n.r	5.3
B3	0.2–0.3	51–70	0.1–55	n.r	n.r	n.r	80
B9	0.7–1	17–26	2.6–7.9	n.r	n.r	n.r	200
B12	0.3–2.4	0.9–1.7	1.6 –3.2	n.r	n.r	0.42	78–195
E	n.r	n.r	n.r	n.r	0.2–1.6	1.5–2	0.2
C	n.r	n.r	n.r	n.r	0.9–1.3	1.8–2.2	0.8
Fatty acids (% total fatty acids)							
C16:0 (palmitic)	20–30	13–41	43–57	11–25	14–16	15–17	19–36
C18:3 n-3 (alpha-linolenic)	22–24	0.9–3	1.3–23	2.4–30	0.1–0.3	22–31	22–28
C18:3 (linolenic)	26–28	0.3–7.4	14–19	22–35	n.r	3.2–3.7	n.r
C16:2 (hexadecadienoic)	12–23	0.1–2	2.2–6	0.4–3.5	1–2.5	12–14	1.8–5
C18:1 (oleic)	29–33	1.6–7.3	1–19	7.6–50	3.7–6.4	5.3–8.9	12.5–20

Information adapted from: ^1^ Ran et al. [12]; Mehariya et al. [13]; Rodrigues-Sousa et al. [14]; ^2^ Ran et al. [15]; Fattore et al. [16]; Nogueira et al. [17]; ^3^ Shanthi et al. [18]; Batista de Oliveira et al. [19]; Morais et al. [20]; ^4^ Xing et al. [21]; Polat et al. [22]; Bohutskyi et al. [23]; ^5^ Jung et al. [24]; Zhu et al. [25]; Kottuparambil et al. [26]; ^6^ Kumudha and Sarada [27]; Mixson Byrd and Burkholder [28]; Torres-Tiji et al. [7]; ^7^ Pereira et al. [29]; Schulze et al. [30]; Qazi et al. [31]. n.r—not reported.

**Table 2:** Studies on the application of microalgal biomass or derivates in yogurt. BFP—before the fermentation process; AFP—after the fermentation process.

Microalgae or Derivate	Addition Rate	Physicochemical, Sensory, Rheology, Textural or Functional Characteristics	References
*Chlorella vulgaris*	0.25, 0.50 and 1% (*w*/*v*) BFP	Final acidity (°D) and final redox potential (mv) were higher than the controls, pH and acetic acid (%) values were not different compared to the controls. Oral texture and feel in the mouth, appearance and nonoral texture were lower than the control.	Beheshtipour et al. [52]
*Isochrysis galbana*	2% (*w*/*w*) AFP	Protein and ash percentages were higher than the controls, lipid content (%) was not different compared to the control. Levels of ω3-fatty acids were higher than the control.	Matos et al. [53]
*Pavlova lutheri*	0.25 and 0.5% (*w*/*v*) AFP	Moisture, carbohydrate, protein and fat contents were not different compared to the control. pH values during storage (28 days) were similar to the control. Addition rate in the treatments was negatively correlated with color, liking of flavor, liking of texture and overall acceptability.	Robertson et al. [54]
Phycocyanin from *Arthrospira platensis*	2, 4 and 8% (*w*/*w*) BFP	Treatments showed pH values higher than the control during 21 days of storage. Supplemented yogurts showed a lower viscosity compared to the control during 21 days of storage. Treatment with 4% of phycocyanin was the most accepted by the panelists.	Mohammadi et al. [55]
*Arthrospira platensis*	0.25, 0.50, 0.75 and 1% (*w*/*v*) BFP	Total solids, protein, ash and fat contents were higher than the control. There was a reduction in pH values of the treatments compared to the control. Fortified samples exhibited lower firmness compared to the control. Yogurts containing 2% of *A. platensis* had the highest score for acceptability.	Barkallah et al. [56]
*Arthrospira platensis*	1.% (*w*/*w*) AFP	Moisture, fat, protein, lactose, and ash levels were higher compared to the control. pH values in fortified samples were greater than the control as well.	Da Silva et al. [51]
*Arthrospira platensis*	0.13, 0.25, 0.38 and 0.5% (*w*/*v*) BFP	Acidity levels in fortified yogurt were greater than the control during 16 days of storage. Overall acceptability decreased with higher amounts of *A. platensis.* The antioxidant capacity was reduced during storage.	Alizadeh et al. [57]
*Arthrospira platensis*	1% (*w*/*w*) BFP	Ash, total solid, fat, and protein contents had an increase compared to the control. There were no significative changes in the acidity and pH values. Total phenolic content and total antioxidant activity were increased in treatments with *A. platensis*. Apparent viscosity values of fortified samples were greater than the control.	Atallah et al. [50]
*Spirulina platensis*	0.1, 0.3 and 0.5% (*w*/*v*) BFP	Solid content, protein, fat, ash, carbohydrate and acidity levels in supplemented yogurts were higher than the control. There was a reduction in the pH values compared to the control. There was an increase in hardness and viscosity values of fortified samples compared to the control.	Bchir et al. [58]

**Table 3:** Studies on the application of microalgal biomass or derivates in ice cream.

Microalgae or Derivate	Addition Rate	Physicochemical, Sensory, Rheological, Textural or Functional Characteristics	References
*Nannochloropsis oculata*	0.1, 0.2 and 0.3% (*w*/*w*)	Fortified samples were greenish in color. There were no changes in the melting behavior of fortified samples. Consistency index (*K*) values of the samples were close to the control.	Durmaz et al. [76]
*Arthrospira platensis*	0.075, 0.15, 0.23 and 0.3% (*w*/*w*)	Acidity in supplemented ice cream was increased compared to the control. pH values of fortified samples were lower than the control sample. Higher amounts of microalgae resulted in a decrease of the viscosity. Overrun in supplemented samples was enhanced compared to control.	Malik et al. [71]
*Arthrospira platensis*	Pure and microencapsulated with maltodextrin or Arabic gum	Protein, fat and total solid were increased in ice cream with microencapsulated or pure *Spirulina* compared to control. Overall acceptability was higher in ice cream without microencapsulated or pure *Spirulina*. Melting time in samples with pure microalgae was lower than samples with microencapsulated *Spirulina*.	Balensiefer et al. [77]
*Arthrospira platensis*	0.6 and 1.2%	Total solid, protein and fat content were increased in enriched ice cream compared to control. Ice cream overrun and melting point were higher in fortified samples. Sensory analysis showed that the panelists preferred ice cream without microalgae.	Agustini et al. [78]
*Diacronema vlkianum*	0.1, 0.2 and 0.3% (*w*/*w*)	Supplemented ice cream was greenish in color. The panelists found a bitter taste in enriched samples. Ice cream with microalgae showed lower *K* values than the control.	Durmaz et al. [76]
Phycocyanin from *Arthrospira platensis*	0.025%	Fortified ice cream was bluish in color (negative values of b*) whereas control samples were yellowish in color (positive values of b*). Antioxidant capacity of supplemented samples was improved after digestion compared to control.	Campos et al. [79]
*Porphyridium cruentum*	0.1, 0.2 and 0.3% (*w*/*w*)	Protein, fat and total solid were increased in ice cream with microencapsulated or pure *Spirulina* compared to control. Phenolic compounds increased with greater amounts of microalgae. A higher quantity of microalgae adversely affected the ice cream general sensory parameters.	Durmaz et al. [76]
Phycocyanin from *Arthrospira platensis*	0.013%	There was no difference in the fat content of supplemented samples compared to control. Melting time in samples with phycocyanin was lower compared to control. Overall acceptability was higher in non-fortified samples.	Rodrigues et al. [80]

**Table 4:** Studies on the application of microalgal biomass in cheeses.

Microalgae	Addition Rate	Physicochemical, Sensory, Rheology, Textural or Functional Characteristics	References
*Chlorella vulgaris*	1, 2 and 3% (*w*/*w*)	There were significant differences between the control and cheese analogue enhanced by 3% *C. vulgaris* biomass in all the chemical components (moisture, fat, carbohydrate and salt content). The microalgae protein and carbohydrates promoted the increase of firmness and the decrease of oil separation indexes of the cheeses.	Mohamed et al. [91]
*Chlorella vulgaris*	2, 4 and 6% (*w*/*w*)	The pH of the cheeses increased with the percentage of microalgae added. The addition of microalgae to the processed cheese increased the degree of meltability compared with the control sample before and after storage.	Tohamy et al. [92]
*Arthrospira platensis*	0.5, 1 and 1.5% (*w*/*w*)	The increase in the amount of microalgae led to a reduction in moisture and an increase in protein and fat content in soft cheese. Cheeses fortified with *Spirulina* showed higher values of β-carotene than then control.	Agustini et al. [78]
*Arthrospira platensis*	0.25, 0.5 and 1% (*w*/*w*)	There was an increase in the protein and fat content in supplemented samples compared to control. Cheeses with 0.25% and 0.5% incorporated *Spirulina* were mostly preferred by the panelists.	Bosnea et al. [93]
*Arthrospira maxima*	1, 2 and 3% (*w*/*w*).	pH of fortified samples decreased slightly compared to the control. Fat, protein and solid total content were increased in samples with 3% of microalgae. Antioxidant capacity was enhanced in supplemented samples at storage compared to the control. Overall acceptability had high scores for all treatments and control.	Mohamed et al. [94]
*Arthrospira platensis*	0.5, 1 and 1.5% (*w*/*w*)	Protein and ash content of enriched cheeses were not affected by microalgae addition. The L* values of *Spirulina*-fortified samples decreased by increasing microalgae concentration. *Spirulina*-fortified samples showed significantly lower degrees of hardness than the control, both at the beginning and end of storage.	Golmakani et al. [95]
*Arthrospira platensis*	0.5, 1 and 1.5% (*w*/*w*)	Fat and protein content of the supplemented cheeses was improved by microalgae addition compared to the control. The addition of microalgae to the cheese increased the phenolic compound and flavonoid content and also the antioxidant capacity.	Mohamed [96]

**Table 5:** Microalgae or derivates approved for adding in milk and dairy products.

Food Authority	Microalgae or Derivate	Food Category	Maximum Levels or Maximum Daily Intake	Additional Specific Labelling Requirements	References
EFSA (EU)	*Arthrospira platensis*	Milk and dairy products	Unlimited ^a^	-	EC 258/97 [114]
	*Chlorella luteoviridis*	Milk and dairy products	Unlimited ^a^	-	EC 258/97 [114]
	*Chlorella pyrenoidosa*	Milk and dairy products	Unlimited ^a^	-	EC 258/97 [114]
	*Chlorella vulgaris*	Milk and dairy products	Unlimited ^a^	-	EC 258/97 [114]
	*Euglena gracilis*	Yogurt and yogurt beverages	0.15 g/100 g	Labelling must content the term “dried biomass of *Euglena gracilis* algae”.	EC 2017/2470 [115]
	*Schizochytrium* sp. oil rich in DHA and EPA	Dairy products except milk-based beverages	0.6 g/100 g for cheese; 0.2 g/100 g for milk products (including milk, fromage frais and yogurt products)	Labelling must content the term “oil from the microalgae *Schizochytrium* sp.”	EC 2017/2470 [115]
	*Ulkenia* sp. Algal oil rich in DHA	Milk-based beverages	0.06 g/100 mL of DHA	Labelling must content the term “oil from the micro-algae *Ulkenia* sp.”	EC 2017/2470 [115]
FDA (USA)	*Dunaliella bardawil*	Low-fat cheese, spreadable cheese and cottage cheese	0.01 g/100 g	-	GNR 276 [116]
	*Arthrospira platensis*	Milk and dairy products	3 g per serving ^b^	-	GNR 417 [116]
	*Euglena gracilis*	Milk and dairy products	0.208 g/100 g for prebiotic and yogurt beverages; 0.222 g/100 g for yogurt	-	GNR 697 [116]
	*Chlorella protothecoides*	Milk and dairy products	1 g/100 g for milk; 1.35 g/100 g for yogurt, cheese and ice cream	-	GNR 519 [116]
	*Chlorella vulgaris*	Milk	1.5 g/100 g	-	GNR 396 [116]
	*Prototheca moriformis* structuring fat	Milk and dairy products	20 g/100 g for butter; 2 g/100 g for milk products	-	GNR 673 [116]
FSSAI (India)	*Chlorella vulgaris*	Milk and dairy products	4 g per day	-	FSSAI [117]
	*Arthrospira platensis*	Milk and dairy products	3 g per day	-	FSSAI [117]
	Phycocyanin from *Arthrospira platensis* dried powder	Milk and dairy products	50 mg per day	-	FSSAI [117]
	Astaxanthin powder from *Haematococcus pluvalis*	Milk and dairy products	4 mg per day	-	FSSAI [117]
ANZFA (Australia and New Zealand)	*Schizochytrium* sp.	Milk products	0.075 g/100 g	-	A428 [118]
	*Schizochytrium* sp. oil rich in DHA	Milk products	260 mg of DHA per day in Australia; 280 mg of DHA per day in New Zealand	-	A428 [118]

^a^ Used prior to May 1997 in EU Novel Food catalog; ^b^ serving sizes based on the USA FDA Reference Amounts Customarily Consumed per Eating Occasion (RACCs) [119].

## Data Availability

Not applicable.
